# Decreased HLA-DR antigen-associated invariant chain (CD74) mRNA expression predicts mortality after septic shock

**DOI:** 10.1186/cc13150

**Published:** 2013-12-10

**Authors:** Marie-Angélique Cazalis, Arnaud Friggeri, Laura Cavé, Julie Demaret, Véronique Barbalat, Elisabeth Cerrato, Alain Lepape, Alexandre Pachot, Guillaume Monneret, Fabienne Venet

**Affiliations:** 1 Joint Research Unit “Sepsis” Hospices Civils de LYON – bioMérieux, Hôpital Edouard Herriot, Lyon, France; 2 Hospices Civils de LYON, Intensive Care Units, Centre Hospitalier Lyon-Sud, Pierre Bénite, France; 3 Hospices Civils de LYON, Immunology Laboratory, Hôpital Edouard Herriot, 5 place d’Arsonval, Lyon 69437, France

## Abstract

**Introduction:**

Septic syndromes remain the leading cause of mortality in intensive care units (ICU). Septic patients rapidly develop immune dysfunctions, the intensity and duration of which have been linked with deleterious outcomes. Decreased mRNA expressions of major histocompatibility complex (MHC) class II-related genes have been reported after sepsis. We investigated whether their mRNA levels in whole blood could predict mortality in septic shock patients.

**Methods:**

A total of 93 septic shock patients were included. On the third day after shock, the mRNA expressions of five MHC class II-related genes (*CD74, HLA-DRA, HLA-DMB, HLA-DMA, CIITA*) were measured by qRT-PCR and monocyte human leukocyte antigen-DR (mHLA-DR) by flow cytometry.

**Results:**

A significant correlation was found among MHC class II related gene expressions. Among mRNA markers, the best prognostic value was obtained for *CD74* (HLA-DR antigen-associated invariant chain). For this parameter, the area under the receiver operating characteristic curve (AUC) was calculated (AUC = 0.67, 95% confidence interval (CI) = 0.55 to 0.79; *P* = 0.01) as well as the optimal cut-off value. After stratification based on this threshold, survival curves showed that a decreased *CD74* mRNA level was associated with increased mortality after septic shock (Log rank test, *P* = 0.0043, Hazard Ratio = 3.0, 95% CI: 1.4 to 6.5). Importantly, this association remained significant after multivariate logistic regression analysis including usual clinical confounders (that is, severity scores, *P* = 0.026, Odds Ratio = 3.4, 95% CI: 1.2 to 9.8).

**Conclusion:**

Decreased *CD74* mRNA expression significantly predicts 28-day mortality after septic shock. After validation in a larger multicentric study, this biomarker could become a robust predictor of death in septic patients.

## Introduction

Despite a drop in mortality rate within recent years, septic syndromes still represent the first cause of death in intensive care units (ICU) [1].

Septic patients develop immune dysfunctions, the intensity and duration of which have been linked with increased risk of death or nosocomial infections [2]. The restoration of normal immune response in patients with immunoadjuvant therapies now emerges as an innovative therapeutic strategy in sepsis [2]. Preliminary clinical trials testing immunostimulating treatments showed promising results [3]. However, as there is no clinical sign of immune dysfunctions in ICU patients, a prerequisite is the development of robust biomarkers easily accessible to clinicians so as to identify those patients who could benefit the most from such therapies.

Among the numerous markers that have been tested for their capacity to predict mortality in septic patients, the decreased expression on circulating monocytes of the major histocompatibility complex (MHC) class II molecule human leukocyte antigen-DR (HLA-DR) measured by flow cytometry (mHLA-DR) has proven to be a reliable predictor of adverse events (death, secondary nosocomial infections) in critically ill patients when measured 48 h after inaugural stress/injury [4]. However, the limited availability of flow cytometers for routine biological analyses has precluded its use in large multicentric clinical evaluations and more generally in everyday clinical practice. Conversely, the ever increasing availability of molecular biology platforms in routine labs should facilitate the standardized use of transcriptomic biomarkers. Although decreased mRNA expressions of MHC class II-related genes have been reported after sepsis [5,6], association with mortality has never been specifically investigated.

In the current study, the capacity of MHC class II-related transcriptomic biomarkers to predict 28-day mortality after septic shock was evaluated.

## Materials and methods

### Study design

The study group consisted of 93 septic shock patients (diagnostic criteria of the American College of Chest Physician/Society of Critical Care Medicine [7]) admitted to the surgical or medical ICUs of Lyon-Sud University hospital and who were alive three days after the onset of shock. The exclusion criteria were patients under the age of 18 years old, subjects with aplasia or immunosuppressive disease (for example, HIV) and patients that died before Day 3. Septic shock was defined by an identifiable site of infection, persisting hypotension despite fluid resuscitation requiring vasopressor therapy, and evidence of a systemic inflammatory response manifested by at least two of the following criteria: a) temperature >38°C or <36°C; b) heart rate >90 beats/minute; c) respiratory rate >20 breaths/minute; d) white blood cell count >12,000/mm^3^ or <4,000/mm^3^. Severity was assessed by the Simplified Acute Physiologic Score II (SAPS II) calculated at inclusion in the protocol. Development of organ dysfunction was assessed by the Sequential Organ Failure Assessment score (SOFA, range: 0 to 24) measured after 24 h of ICU stay. Mortality was defined as death occurring within 28 days after the onset of shock. The onset of septic shock was defined as the beginning of vasopressor therapy. In accordance with guidelines from the Surviving Sepsis Campaign and from our ICUs, septic shock patients were rapidly treated with empiric broad-spectrum antibiotherapy after admission.

Biological analyses were performed on residual blood after completing routine follow-up performed in the ICU. PAXgene® and EDTA-anti-coagulated tubes were collected at the same time from patients at Day 3 or Day 4 after diagnosis of septic shock. This work belongs to a global study on ICU-induced immune dysfunctions. It has been approved by our Institutional Review Board for ethics (“Comité de Protection des Personnes”) which waived the need for informed consent because this study was observational and biomarker expressions were measured on a very low volume of residual blood after completion of routine follow-up. This study is also registered at the French Ministry of Research and Teaching (#DC-2008-509) and recorded at the Commission Nationale de l’Informatique et des Libertés.

### Flow cytometry

Circulating monocyte HLA-DR expression (mHLA-DR) was assessed by flow cytometry (NAVIOS; Beckman-Coulter, Miami, FL) as previously described [8]. Results are expressed as the number of antibodies bound per cells (AB/C).

### RNA extraction, reverse transcription and quantitative PCR

Whole-blood mRNA expressions of five MHC-class II related genes (*HLA-DRA, HLA-DMA, HLA-DMB, CIITA, CD74*) were studied using blood samples collected directly in PAXgene blood RNA tubes (PreAnalytix, Hilden, Germany). Total RNA was extracted using the PAXgene Blood RNA kit (PreAnalytix). Before RNA elution, the residual genomic DNA was digested using the Rnase-Free Dnase set (Qiagen, Hilden, Germany). Total RNA was reverse transcribed into complementary DNA (cDNA) using SuperScript® VILO TM cDNA Synthetis (Life Technologies, Grand Island, NY). The gene panel constituting the MHC II was quantified using q- real time polymerase chain reaction. Polymerase chain reaction was performed in a LightCycler instrument using the standard Taqman Fast Advanced Master Mix PCR kit according to the manufacturer’s instructions (Roche Molecular Biochemicals, Basel, Switzerland). Thermocycling was performed in a final volume of 20 μL containing 5 μM of the required primers, 1 μM of required probe. PCR was performed with an initial denaturation step of 10 minutes at 95°C, followed by 45 cycles of a touchdown PCR protocol (10 sec at 95°C, 29 sec annealing at 68°C, and 1 sec extension at 72°C). The cDNA standards were prepared from purified PCR amplicons obtained with the corresponding primers (Table 1). The Second Derivative Maximum Method was used with the LightCycler software to automatically determine the crossing point for individual samples as previously described [1]. Standard curves were generated by using the quadruplicate cDNA standard. Relative standard curves describing the PCR efficiency of selected genes were created and used to perform efficiency-corrected quantification with the LightCycler Relative Quantification Software (Roche Molecular Biochemicals). Gene expression normalization was performed based on the combination of two selected housekeeping genes (*HPRT1: hypoxanthine phosphoribosyltransferase 1 and GLYR1: glyoxylate reductase 1 homolog*) and results were expressed as Calibrated Normalized Relative Quantity (CNRQ) [2]. Both reference genes (*HPRT1* and *GLYR1*) were selected based on an ongoing project on reference genes usable in genomic studies in ICU patients. These were selected among a list of other reference genes based on their stability and the absence of differential expression between compared groups of patients. This analysis was performed by using the tools available via [9] (data not shown).

**Table 1:** Primer designs

**Gene**	**Accession no.**	**Sequence**
*HLA-DRA*	NM_019111	5′-GATGCTCCAAGCCCTCTCCCAG-3′ (probe) (22)
		5′-GCCTCTTCTCAAGCACTGGGA-3′ (sense) (21)
		5′-CCACCAGACCCACAGTCAGG-3′ (antisense) (20)
*HLA-DMA*	NM_006120	5′-TCCCTGAAGCTCCTACTCCAA-3′ (probe) (22)
		5′-CTGTGTGGCAAGAAGGTATG-3′ (sense) (20)
		5′-TCCTGGCAGTACACTGTGT-3′ (antisense) (19)
*HLA-DMB*	NM_002118	5′-GAGCAGGTGGCTTCGTGGC-3′ (probe) (19)
		5′-CATCTTTACAGAGCAGAGCAT-3′ (sense) (21)
		5′-ATGTGAAATCCTTTGGAGTCC-3′ (antisense) (21)
*CIITA*	NM_000246	5′-CTCAGAACCCGACACAGACAC-3′ (probe) (21)
		5′-CCTGGCTGGAGAAGAAGAG-3′ (sense) (19)
		5′-TCCTGGAAGACATACTGGTC-3′ (antisense) (20)
*CD74*	NM_004355	5′-CCAGCGAGGAGCAGAGTCAC-3′ (probe) (20)
		5′-TTATCTCCAACAATGAGCAACT-3′ (sense) (22)
		5′-ACAGGAAGTAGGCGGTGGT-3′ (antisense) (19)
*HPRT1*	NM_000194.2	5′-CAAGTTTGTTGTAGGATATGCCC-3′ (probe) (23)
		5′-CCAAAGATGGTCAAGGTCGC-3′ (sense) (20)
		5′-GACACAAACATGATTCAAATCC-3′ (antisense) (22)
*PPIB*	NM_000942.4	5′-GGTGAGCATGGCCAACGCAGG-3′ (probe) (22)
		5′-GGAGATGGCACAGGAGGAAAGA-3′ (sense) (22)
		5′-GGGAGCCGTTGGTGTCTTTG-3′ (antisense) (20)

Design of primers used for the messenger RNA quantification of major histocompatibility class II related genes and selected housekeeping genes using real-time polymerase chain reaction. CD74, HLA-DR antigen-associated invariant chain; CIITA, class II transactivator; HLA-DR/DM, human leukocyte antigen-DR/DM; HPRT1, hypoxanthine phosphoribosyltransferase 1; PPIB, peptidylprolyl isomerase B (cyclophilin B).

### Statistical analysis

Comparisons between groups were made using the non-parametric Mann Whitney U-test (survivors *vs* non-survivors) for continuous variables and the Pearson chi-squared test, as appropriate, for categorical data. Correlation studies were performed with the Spearman’s rank correlation test. Receiver Operating Curves were performed to determine the cut-off values for *CD74* mRNA or mHLA-DR expressions in regard to prediction of mortality. The best cut-off value was selected based on optimized Youden index. Using these thresholds, Kaplan-Meier survival curves were obtained and differences in survival between groups were evaluated using Log rank test. Uni- and multi-variate logistic regression analyses were used to identify the variables associated with death and Cox model permitted to estimate the Hazard Ratio and 95% confidence interval (CI 95%). Backward selection was used and a *P*-value of 0.05 was considered as statistically significant. Because SAPS II and SOFA scores confirmed the assumed linearity with the outcome (survivors *vs* non survivors), these variables were included in the model as continuous variables. Statistical analyses were performed with SPSS (version 17.0, SPSS, Chicago, IL, USA) and GraphPad Prism® (version 4.03, GraphPad Software, La Jolla, CA, USA) softwares.

## Results

Ninety-three septic shock patients were included in this study. Only patients alive and sampled at Day 3 or Day 4 after the onset of shock were included in this cohort as previous studies showed that the mHLA-DR predictive value on deleterious outcomes (death, nosocomial infections) after septic shock is maximal at this time point [4]. Clinical and biological characteristics for this cohort are listed in Table 2. Twenty-seven patients died within 28 days (mortality = 29%). Median time of death was nine days after the onset of shock (Q1 to Q3: 5 to 18 days). The mRNA levels of five MHC class II-related genes (*HLA-DRA, HLA-DMA, HLA-DMB, CIITA* and *CD74*) as well as mHLA-DR were evaluated in this cohort.

**Table 2:** Demographic and clinical data for septic shock patients

	**Survivors n = 66 (%)**	**Non-survivors n = 27 (%)**	***P*-value**	**Total n = 93 (%)**
**Male**	44 (67)	16 (59)	ns	60 (65)
Age*	61 [53 to 73]	65 [55 to 77]	ns	62 [54 to 75]
SAPS II on admission*	45 [34 to 54]	57 [46 to 69]	<0.0001	48 [35 to 60]
Duration length in ICU*	25 [8 to 37]	12 [5-17]	0.0074	21 [6 to 31]
SOFA*	9 [7-11]	12 [9-12]	0.0013	10 [8-12]
**Co-morbidities**			<0.001	
0	44 (65.7)	4 (15.4)		48 (51.61)
1	19 (28.4)	16 (61.5)		35 (37.6)
>2	4 (5.9)	6 (23.1)		10 (10.8)
**Type of admission**			ns	
Surgery	30 (46)	10 (37)		40 (43)
Medical	36 (54)	17 (63)		53 (57)
**Type of infection**			ns	
Community acquired	32 (49)	18 (67)		50 (54)
Nosocomial	34 (51)	9 (33)		43 (46)
**Suspected infection**			ns	
Clinically documented	9 (14)	3 (11)		12 (13)
Microbiologically	55 (83)	22 (85)		78 (84)
Documented				
-Bacilli Gram (−)	35 (53)	12 (44)		47 (51)
-Cocci Gram (+)	23 (35)	11 (41)		34 (37)
-Fungi	14 (21)	8 (30)		22 (23)
**Primary site of infection**			0.0015	
-Abdominal	23 (35)	8 (30)		31 (33)
-Pulmonary	27 (41)	15 (55)		42 (43)
-Other	0 (0)	4 (15)		4 (4)

Blood samples were obtained from 93 septic shock patients at Day 3 or Day 4 after the onset of shock. Categorical data are presented as number of cases and percentages respective to the corresponding patient’s population between brackets. Continuous data (*) are presented as medians and interquartile ranges [Q1 to Q3]. SAPS II, Simplified Acute Physiologic Score II calculated at inclusion in the protocol; SOFA score, Sequential Organ Failure Assessment score measured after 24 h of ICU stay. Comparisons between groups (survivors *vs* non-survivors) were made using the non-parametric Mann Whitney U-test for continuous variables and the Pearson chi-squared test, as appropriate, for categorical data. Most frequent co-morbidities included chronic respiratory failure (n = 10 non-survivors and 4 survivors), chronic cardiac failure (n = 3 non-survivors and 4 survivors), chronic renal failure (n = 3 non-survivors and 5 survivors) and metastatic cancer (n = 3 non-survivors and 5 survivors). Other co-morbidities included hematologic malignancies, type II diabetes and chronic hepatic failure.

We first observed that whole blood mRNA levels of these MHC class II genes were highly correlated (Table 3). Indeed, Spearman’s rank correlation coefficients ranged from 0.85 to 0.92 illustrating a strong correlation between MHC class II gene expressions in our cohort. A modest but significant correlation was also observed between these MHC class II gene levels and mHLA-DR measured by FACS. The highest correlation was found with the invariant chain, that is, *CD74*, mRNA expression (r = 0.54; *P* <0.0001, Spearman correlation test, Table 3).

**Table 3:** Correlations

	***HLA-DMB***	***HLA-DMA***	***CD74***	***CIITA***	***HLA-DRA***
**mHLA-DR**	0.52	0.51	0.54	0.49	0.53
***HLA-DMB***		0.88	0.89	0.85	0.92
***HLA-DMA***			0.87	0.87	0.92
***CD74***				0.88	0.87
***CIITA***					0.87

Major histocompatibility class II-related genes mRNA levels and circulating monocyte HLA-DR expression (mHLA-DR) were measured in blood samples obtained from 93 septic shock patients at Day 3 or Day 4 after the onset of shock. Correlation coefficients are shown (Spearman’s rank correlation test). CD74, HLA-DR antigen-associated invariant chain; CIITA, class II transactivator; HLA-DR/DM, human leukocyte antigen-DR/DM.

**Figure 1:**
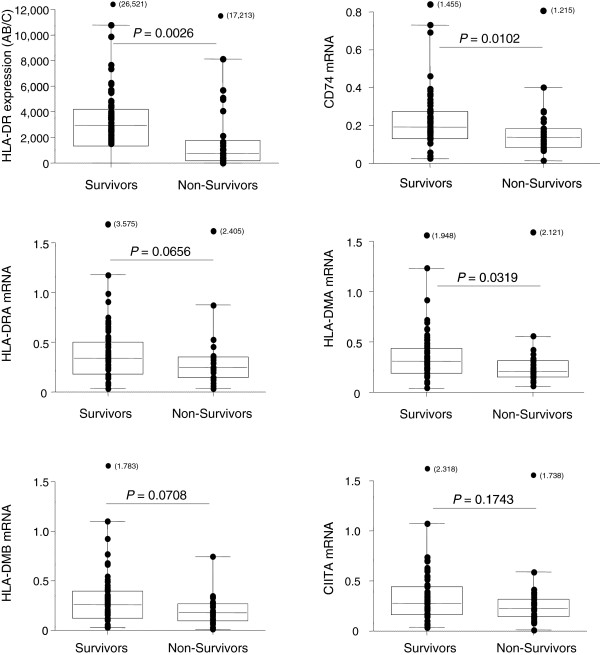
**MHC class II related genes and protein expressions in survivors and non-survivors after septic shock.** Circulating monocyte HLA-DR expression mHLA-DR – number of antibodies bound per cell (AB/C) was measured by flow cytometry and major histocompatibility class II related genes mRNA levels were evaluated by qRT-PCR in blood samples obtained from 93 septic shock patients at Day 3 or Day 4 after the onset of shock. Results are presented as box-plots as well as individual values in survivors (n = 66) and non-survivors (n = 27). Mann Whitney U-test was used to compare results between groups. CD74, HLA-DR antigen-associated invariant chain, CIITA, class II transactivator; HLA-DR/DM, human leukocyte antigen-DR/DM.

These biomarkers’ mRNA expressions were subsequently evaluated in survivors *vs* non-survivors. Non-survivors presented with significantly lower mHLA-DR (*P* = 0.0026, Mann Whitney U-test, Figure 1). In this cohort, among MHC class II-related genes, only *CD74* and *HLA-DMA* mRNA levels were significantly decreased in non-survivors (*P* = 0.0102 and *P* = 0.0319, respectively, Mann Whitney U-test, Figure 1).

**Figure 2:**
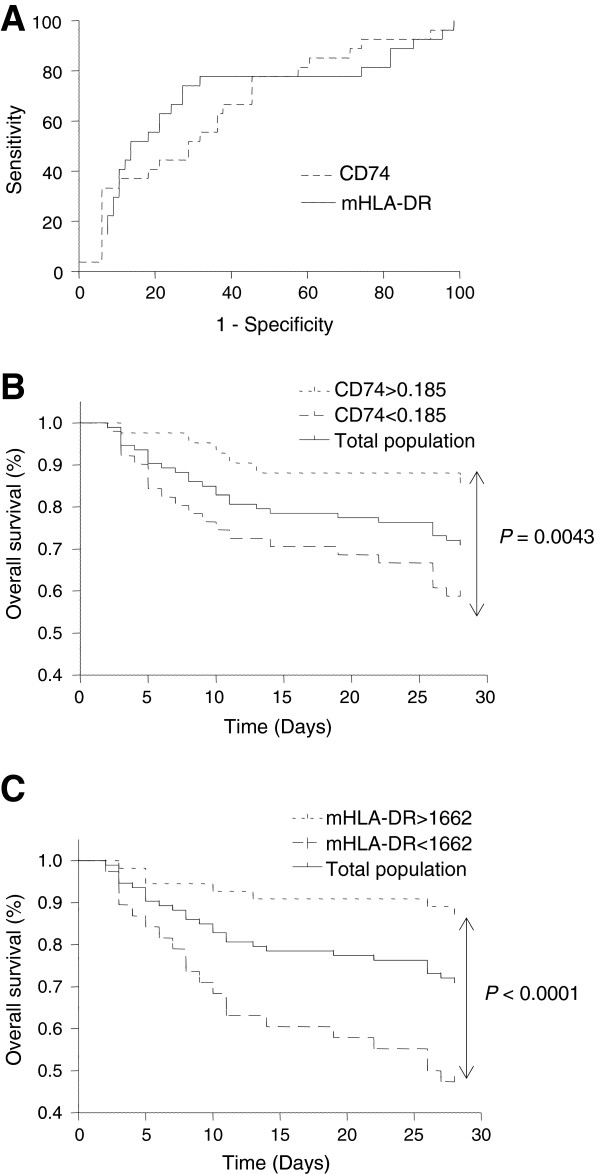
**Prognostic value of mHLA-DR and *CD74 *mRNA levels in septic shock patients.***CD74* mRNA level and circulating monocyte HLA-DR expression (mHLA-DR) were measured in blood samples obtained from 93 septic shock patients at Day 3 or Day 4 after the onset of shock. **A)**. Receiver operating characteristic curves were established for these two parameters. The area under the curve was 0.67 for *CD74* (95% confidence interval (95% CI): 0.55 to 0.79; *P* = 0.01) and 0.70 (95% CI: 0.57 to 0.83; *P* = 0.003) for mHLA-DR. **B)**. Kaplan-Meier survival curves were established after stratification based on *CD74* mRNA level cut-off value (= 0.185, optimized Youden index). A significant difference was measured between the two curves (Log rank test, *P* = 0.0043; Hazard Ratio = 3.0, 95% CI: 1.4 to 6.5). **C)**. Similar analysis was performed for mHLA-DR. Cut-off value = 1,662 AB/C. Log rank test: *P* <0.0001, Hazard Ratio = 5.8, 95% CI = 2.6 to 12.9.

The areas under the receiver operating characteristic curves (AUC) for prediction of 28-day mortality were subsequently calculated for mHLA-DR and *CD74*. The AUC was 0.67 for *CD74* (95% confidence interval (95% CI): 0.55 to 0.79; *P* = 0.01) and 0.70 (95% CI: 0.57 to 0.83; *P* = 0.003) for mHLA-DR (Figure 2A). Cut-off values were then determined based on calculation of the optimized Youden index. Using this cut-off (that is, 0.185), 21 out of 27 non-survivors could be identified based on a decreased *CD74* mRNA expression. To note, among the six non-survivors that presented a *CD74* mRNA level higher than this calculated threshold, four also had a mHLA-DR value higher than its respective cut-off value (that is, 1,662).

Kaplan-Meier survival curves were established after stratification based on calculated thresholds (Figure 2B-C). The survival rates of patients were significantly different when stratified according to *CD74* mRNA expression. Indeed, patients with higher *CD74* mRNA levels had a significantly better survival compared with patients with lower expression (*P* = 0.0043, Log rank test, Hazard Ratio = 3.0, 95% CI: 1.4 to 6.5, Figure 2B). Similarly, the survival rate of patients was significantly different when the cohort was stratified based on mHLA-DR expression. Patients with mHLA-DR >1,662 presented with a better survival compared with patients with decreased mHLA-DR (*P* <0.0001, Log rank Test, Hazard ratio = 5.8, 95% CI = 2.6 to 12.9, Figure 2C).

Logistic regression analyses were finally performed to assess if these markers remained independently associated with mortality after septic shock when combined with usual clinical confounders. Importantly, in such multivariate analysis, including SOFA and SAPS II scores, decreased *CD74* mRNA level (Odds Ratio = 3.4, 95% CI: 1.2 to 9.8, *P* = 0.026) or diminished mHLA-DR (Odds Ratio = 7.8, 95% CI: 2.6 to 23.4, P <0.001) remained significantly associated with a higher risk of death after septic shock (Table 4).

**Table 4:** Multivariate analysis

	**Univariate**	**Multivariate**	
	***P***	**Odds ratio (95% CI)**	***P***
**mHLA-DR**	<0.0001	7.8 (2.6 to 23.4)	<0.0001
**SOFA**	0.011	3.3 (1.1 to 10.2)	0.034
**SAPS II**	0.013	1.7 (0.6 to 5.1)	0.341
***CD74***	0.006	3.4 (1.2 to 9.8)	0.026
**SOFA**	0.011	2.3 (0.8 to 6.4)	0.106
**SAPS II**	0.013	2.1 (0.7 to 5.8)	0.166

Major histocompatibility class II-related gene mRNA levels and circulating monocyte HLA-DR expression (mHLA-DR) were measured in blood samples obtained from 93 septic shock patients at Day 3 or Day 4 after the onset of shock. Uni- and multi-variate logistic regression analyses were used to identify the variables associated with death (n = 66 survivors and 27 non-survivors) and Cox model permitted to estimate the Hazard Ratio and 95% confidence interval (CI 95%). Backward selection was used and a *P*-value of 0.05 was considered as statistically significant. Because SAP II and SOFA confirmed the assumed linearity with the outcome (survivors vs non-survivors), these variables were included in the model as continuous variables. CD74, HLA-DR antigen-associated invariant chain; HLA-DR, human leukocyte antigen-DR/DM; SAPS II, Simplified Acute Physiologic Score II calculated at inclusion in the protocol; SOFA score, Sequential Organ Failure Assessment score measured after 24 h of ICU stay.

## Discussion

Despite improvement in patients’ care, septic syndromes remain a public health challenge [1]. Indeed, a recent epidemiological study including more than 25,000 patients in the USA and in Europe showed that overall hospital mortality from severe sepsis or septic shock is still over 30% [10]. In line with this, one in 1,200 Americans will die of severe sepsis this year and this is despite numerous clinical trials testing various therapies and including thousands of patients [11].

After severe sepsis, the development of a state of immune suppression is now a well-documented phenomenon [2,12]. This immunoparalysis is obviously associated with the occurrence of fatal outcome and in lowering patients’ resistance to secondary infections. It is now hypothesized that restoring immune functions in ICU patients could represent a major therapeutic avenue [13]. Nevertheless, a crucial aspect is our capacity to identify only the most immunosuppressed patients (that is, who could benefit the most from immunostimulating therapies).

In this context, the need for biomarkers of host response to infection helping the clinicians to 1) identify the most severe patients (at risk of developing secondary nosocomial infection); 2) stratify patients before the initiation of targeted therapy; and 3) to follow response to treatment are mandatory if we are to decrease mortality from this major public health challenge [11].

Numerous markers of immune failure have been described in patients. Among these, the decreased expression on circulating monocytes of HLA-DR measured by flow cytometry has repeatedly been described as a robust marker of immune dysfunctions in septic shock patients [4].

**Figure 3:**
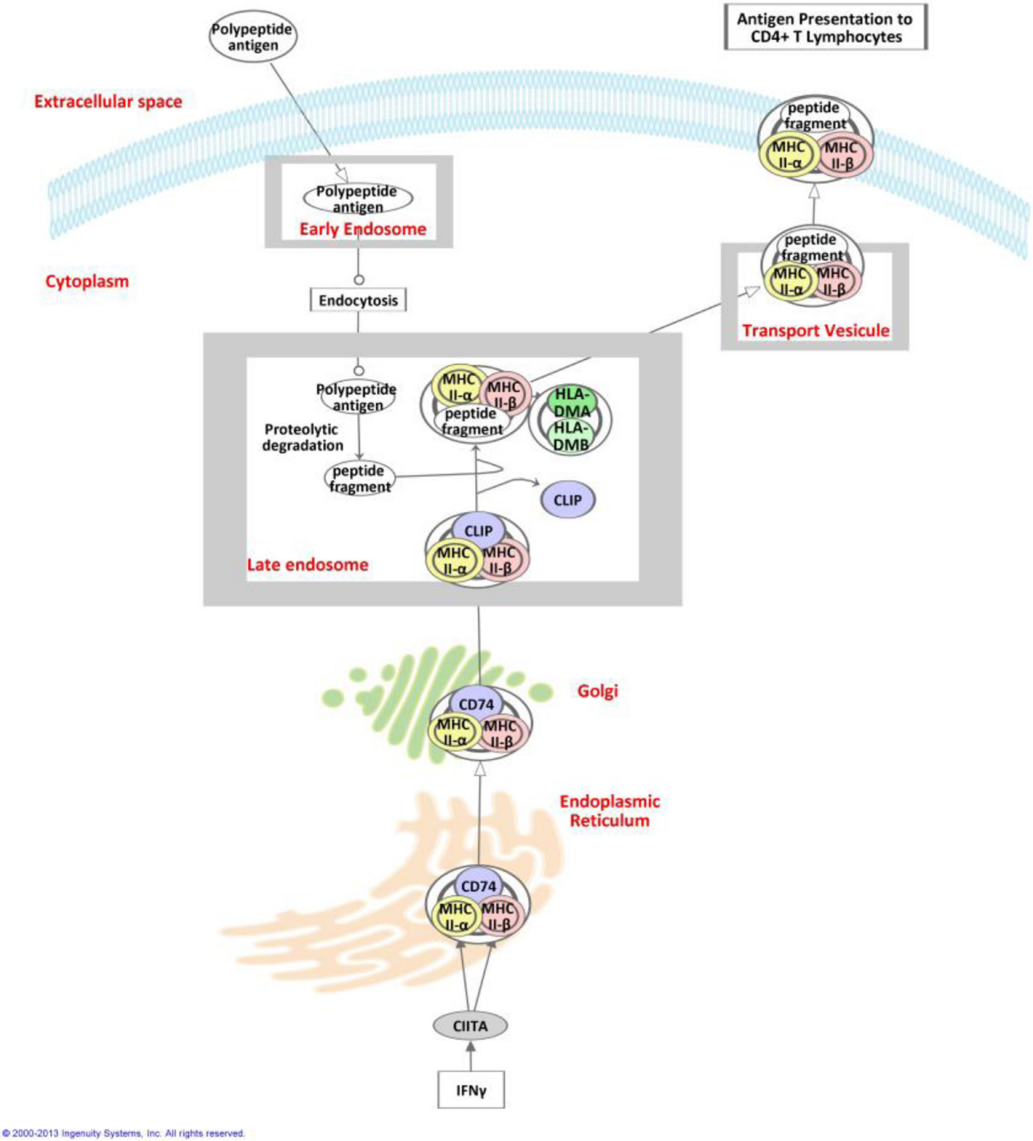
**Major histocompatibility class II related molecules and antigen presentation process.** The MHC class II network was generated through the use of IPA (Ingenuity® Systems, http://www.ingenuity.com).

The MHC class II molecules, including HLA-DR, are heterodimers constituted of an α and a β chain (MHC II-α or HLA-DRA and MHC II-β or HLA-DRB - Figure 3) and are mainly expressed at the surface of antigen-presenting cells [14]. MHC class II gene transcription is under the control of class II transactivator A (CIITA) and factors of the Regulatory Factor X family. During their synthesis, the α and β chains are complexed with the invariant chain (that is, CD74 or Ii), therefore preventing the binding of newly synthesized MHC II α/β heterodimers with the numerous polypeptides that are normally present in the endoplasmic reticulum (ER). CD74 also facilitates the export of class II MHC from the ER to the Golgi, and then to the late endosome compartment. During this stage, CD74 is broken down in stages by proteases called cathepsins, leaving only a small fragment known as CLIP (class II associated invariant chain peptide), which maintains blockage of the peptide binding cleft on the MHC molecule. Peptides derived from extracellular proteins that are endocytosed, ingested in lysosomes are then loaded with the class II MHC molecule prior to the molecule’s migration to the cellular membrane. During this process, an MHC class II-like structure, HLA-DM (heterodimer of HLA-DMA and HLA-DMB), facilitates CLIP removal and allows the binding of peptides with higher affinities. The stable class II MHC is then presented on the cell surface [14].

In terms of functionality, monocytes with decreased mHLA-DR have been shown to be unable to mount a pro-inflammatory response to any bacterial challenge or to properly present antigens to T cells [4]. In terms of clinical information, decreased mHLA-DR has been shown to be predictive of both fatal outcome and septic complications after trauma, surgery, pancreatitis, burn and septic shock [4]. Recently, this parameter has been used to stratify the administration of GM-CSF in a clinical trial including a small cohort of septic patients [3]. This biomarker-guided GM-CSF therapy appeared safe and effective in restoring monocyte immunocompetence and shortening the duration of mechanical ventilation and hospital/ICU stay. Therefore, the next challenge would be to design a multicentric clinical trial testing this biomarker-guided approach in a large cohort of patients.

However, although an internationally accepted standardized protocol has been designed [15], the measurement of mHLA-DR by flow cytometry remains not easily accessible to clinicians. In particular, strict pre-analytical conditions (delay between sampling and measurement) have to be respected [16]. Moreover, and most importantly, flow cytometers are not often available 24/7 in hospitals.

Conversely, with the development of rapid molecular techniques for diagnosis of infection, numerous mRNA measuring platforms are being developed and becoming available in hospitals. This would most likely permit the concomitant development of transcriptomic biomarkers of host response to infection. We, therefore, asked whether the measurement of MHC class II-related genes could predict 28-day mortality after septic shock. In a cohort of 93 septic shock patients alive three days after shock, mHLA-DR was measured by flow cytometry and *CD74, HLA-DRA, HLA-DMA, HLA-DMB *and *CIITA* mRNA levels were evaluated in whole blood by qRT-PCR.

We observed that among these five MHC class II-related genes, the invariant chain (*CD74*) mRNA was the best predictor of 28-day mortality after septic shock. Similarly to our results, several studies investigated the transcriptional regulation of MHC class II gene expressions in ICU patients. For example, Le Tulzo *et al.* described in a small group of septic patients, a decrease in circulating *HLA-DR* mRNA levels and a relationship between *HLA-DR* mRNA and protein expressions in this cohort. In particular, a strong correlation between mHLA-DR and *CIITA* and *HLA-DR* mRNA levels was shown [17]. Similarly, *CD74* expression was previously noted to be markedly decreased in the early phase of human septic shock either in our group and by other investigators [5,6] and a parallel recovery of *CD74* mRNA and HLA-DR protein expressions was observed in septic patients [6]. However, none of these studies was specifically designed to investigate predictive value of these transcriptomic biomarkers in regard to mortality after sepsis.

In this respect, we observed for the first time that, among MHC class II genes, a decreased *CD74* mRNA level presented with the best predictive value in regard to 28-day mortality after septic shock. Importantly, survival curves showed that patients with low *CD74* mRNA level presented with a significantly higher risk of death than patients with high *CD74* expression. Importantly, this predictive capacity was maintained after multivariate analyses, including usual clinical confounders (SAPS II and SOFA scores). This suggests that, after validation in a larger multicenter study, the evaluation of *CD74* mRNA level could become a robust predictor of mortality in septic patients.

Moreover, in addition to other studies, these results highlight the potential of using transcriptomic biomarkers in the clinic [18]. The utility of such biomarkers lies in their capacity to provide timely information beyond that which is readily available from routine physiologic data and clinical examination. This additional information may provide insight into the pathogenesis or prognosis of a disease process and also aid in a therapeutic decision [19]. Moreover, they may facilitate titrating therapy or monitoring the response to intervention [20]. This is all the more because of the availability in routine labs of molecular biology platforms that will enable the standardized and routine monitoring of such biomarkers.

Our study has some limitations. First, beside mHLA-DR measurement, no specific evaluation of septic patients’ immune functionality was performed in parallel with *CD74* mRNA level. Therefore, we cannot conclude from the current results that a decreased *CD74* mRNA expression is a marker of immunosuppression in sepsis. This aspect needs to be evaluated in a further study. Second, the evaluation of MHC class II-related gene expressions in whole blood as opposed to purified cells precludes us from determining the specific cell type presenting with these decreased mRNA levels. This could also partly explain the modest correlation observed between MHC class II-related genes mRNA levels and mHLA-DR protein expression measured by flow cytometry. In line, different cell types in circulating whole blood express HLA-DR (monocytes, dendritic cells, B lymphocytes and activated T lymphocytes) which may have different intensities and kinetics of MHC class II expression regulation. While most studies have focused on monocytes, very few data are available regarding the regulation of HLA-DR expression on these other cell types after septic shock. In addition, as protein vs mRNA markers were measured, this low correlation could be explained by post-translational regulation mechanisms or differences in kinetics of expression (mRNA being expressed before the protein). In addition, our study was not designed to specifically investigate the mechanisms leading to decreased mHLA-DR expression after septic shock (translational or post-translational). Moreover, only patients alive at Day 3 were included in the study. Therefore, biomarkers of fulminant septic shock were not evaluated here. Since severity scores were measured at admission, the design of this study may have been in favor of a better prognostic value of our markers *vs* these scores. Finally, this study was not designed to directly compare the performances of *CD74* mRNA level *vs* mHLA-DR in predicting 28-day mortality after septic shock but to identify the most promising prognostic marker among several MHC class II related genes. Obviously, a dedicated study is now mandatory to confirm these preliminary results.

## Conclusions

In total, these results show that decreased invariant chain/*CD74* mRNA expression significantly predict 28-day mortality in septic shock patients alive at Day 3. After validation in a large multicenter study, this biomarker could become a robust predictor of death in septic patients.

## Key messages

•Decreased HLA-DR antigen-associated invariant chain (*CD74*) mRNA expression significantly predicts 28-day mortality three days after septic shock.

•Upon validation in a large multicentric study, this biomarker could become a robust predictor of death in septic patients and a relevant tool to monitor patients’ immune status.

## Abbreviations

AB/C: Antibodies bound per cell; AUC: Area under the receiver operating characteristic curve; CI: Confidence interval; CLIP: Class II associated invariant chain peptide; CIITA: Class II transactivator; MHC: Major histocompatibility complex; mHLA-DR: Monocyte human leukocyte antigen-DR; SAPS II: Simplified Acute Physiologic Score II; SOFA: Sequential Organ Failure Assessment score.

## Competing interests

This study was supported by Hospices Civils de LYON and bioMérieux within the context of the Joint Research Unit HCL-bioMérieux in Hôpital E Herriot - Lyon. MAC, VB, EC and AP are bioMérieux’s employees. FV, AF, LC, JD, AL and GM declare that they have no competing interests.

## Authors’ contributions

MAC, LC, VB and EC performed the experiments (molecular biology). JD performed the experiments (flow cytometry). FV and MAC wrote the paper and designed the experiments. FV, AF, AL, AP and GM analyzed and discussed the results, and read and discussed the paper. MAC and JD performed the statistical analyses. All authors read and approved the final manuscript.
